# Climate Change Impacts on Plant Phenology: Grapevine (*Vitis vinifera*) Bud Break in Wintertime in Southern Italy

**DOI:** 10.3390/foods10112769

**Published:** 2021-11-11

**Authors:** Daniel Grigorie Dinu, Valentina Ricciardi, Cosimo Demarco, Gianroberto Zingarofalo, Gabriella De Lorenzis, Riccardo Buccolieri, Gabriele Cola, Laura Rustioni

**Affiliations:** 1 Dipartimento di Scienze e Tecnologie Biologiche ed Ambientali, Università del Salento, S.P. 6 Lecce-Monteroni, 73100 Lecce, Italy; daniel.dinu@unisalento.it (D.G.D.); cosimo.demarco@studenti.unisalento.it (C.D.); gianroberto.zingarofalo@studenti.unisalento.it (G.Z.); Riccardo.Buccolieri@unisalento.it (R.B.); 2 Department of Agricultural and Environmental Sciences, Università degli Studi di Milano, Via Celoria, 20133 Milano, Italy; valentina.ricciardi@unimi.it (V.R.); gabriella.delorenzis@unimi.it (G.D.L.); gabriele.cola@unimi.it (G.C.)

**Keywords:** viticulture, winter warming, biodiversity preservation, cultivar selection, adaptation strategy, frost risks

## Abstract

The effects of global warming on plants are not limited to the exacerbation of summer stresses; they could also induce dormancy dysfunctions. In January 2020, a bud break was observed in an old poly-varietal vineyard. Meteorological data elaboration of the 1951–2020 period confirmed the general climatic warming of the area and highlighted the particular high temperatures of the last winter. Phenological records appeared to be significantly correlated to wood hydration and starch reserve consumption, demonstrating a systemic response of the plant to the warm conditions. The eight cultivars, identified by single-nucleotide polymorphism (SNP) profiles and ampelographic description, grown in this vineyard showed different behaviors. Among them, the neglected Sprino, Baresana, Bianco Palmento, and Uva Gerusalemme, as well as the interspecific hybrid Seyve Villard 12.375, appeared to be the most interesting. Among the adaptation strategies to climate changes, the cultivar selection should be considered a priority, as it reduces the inputs required for the plant management over the entire life cycle of the vineyard. Hot Mediterranean areas, such as Salento, are a battlefront against the climate change impacts, and, thus, they represent a precious source of biodiversity for viticulture.

## 1. Introduction

Viticulture, and more generally agriculture, has to always deal with the variability of environmental conditions and with the effects of climate changes. The increase in temperature that has characterized the last 30 years in Europe [1,2,3] has affected the viticultural sector in different ways and cannot be approached in a generalized way. For example, in the temperate zone, climate change could lead to positive effects on grapevine production [4]. Nevertheless, different responses should be expected in each viticultural area, as highlighted by Santos et al. [5] and Malheiro et al. [6] in their studies concerning the current and future effects of climate change over Europe. Van Leeuwen and Darriet [7] stated that “depending on the region and the amount of change, this may have positive or negative implications on wine quality. Adaptation strategies are needed to continue to produce high-quality wines and to preserve their typicity according to their origin in a changing climate”.

Generally, plant research is mainly focused on the active growth (vegetative and reproductive) of plants and their relationship with environmental stresses (such as drought, heat waves, sunburn). However, more attention should be paid to the tree’s dormancy period as well. In this context, the higher availability of thermal resources during early spring can determine an early start to vegetative growth, increasing the risk for frost damages [8,9,10]. Grapevine, a polycarpic woody plant, develops axillary buds with embryonic shoots from which it forms complete branches after perceiving precise signals. To elude adverse environmental conditions, these buds become dormant, providing the possibility to resume growth under viable conditions. Three dormancy states have been distinguished in buds: paradormancy (induced by distal organs during the vegetative period); endodormancy (to avoid budbreak in autumn and to prepare plants for freezing temperatures; it is characterized by growth inhibition, arrest of cell division, and reduced metabolic and respiratory activity); and ecodormancy (generally related to low temperatures, it is due to unsuitable environmental conditions for active bud growth) [11,12,13,14,15,16,17,18].

Londo and Martinson [19] discussed the grapevine winter survival and the effects of warmer climate on the earlier satisfaction of chilling requirement related to endodormancy and on the earlier end of ecodormancy. In Italian conditions, endodormancy is always satisfied very quickly, and, as a consequence, warm temperatures during winter could produce a phenological anticipation and an early budburst, increasing the risk of frost. These events could be exacerbated in already hot Mediterranean areas, such as Salento (Apulia, South Italy).

Frost susceptibility depends on many factors, including cultivars, pre-freeze environmental conditions (dew point and the presence of surface moisture), the probability of ice nucleation events, and the stage of phenological development [14]. Frost tolerance results from variations in carbon and water balances. Under current climatic conditions, frost risks are greatest for buds in autumn or spring, and for stems in autumn when acclimation is delayed [16]. The risk of frost damage increases significantly as bud development progresses, and once the growth stage of “cotton buds” has passed, buds become more sensitive to freezing, and the consequent damages become more extensive [20]. Late or relatively light freeze events may damage flowers or inflorescences on shoots, whereas early or severe freeze events can kill the whole primary shoot. Injury to flowers, leaves, and shoots has the potential to affect yield not only in the current season of the freeze event, but also in the following season [14]. Damaged tissues rapidly lose their turgor, darken completely, and become water-soaked. After budburst, the damaging temperatures range from −0.5 to −1 °C, and new leaves and shoots become susceptible to temperatures only slightly below 0 °C [21].

Researchers have already proposed vineyard management techniques able to postpone budding: delaying winter pruning until after budburst, the development of basal nodes is inhibited, delaying the budburst on the resulting spurs as well [22].

Late winter pruning (within the stage of two unfolded leaves) and double-pruning techniques affect both yield and grape quality [23,24,25,26,27]. It has been demonstrated that an excessive pruning delay may lead to significant yield losses, and it produces musts characterized by lower sugar contents and higher acidity [25,28,29,30,31,32,33]. However, it worth noting that a delay in the plant phenology could also displace grape ripening processes towards less warm periods [14,32]. In fact, in regions with temperate and semiarid climates, global warming is advancing grapevine ripening, causing an increase in must pH and affecting the phenolic and aromatic compounds [32].

These strategies are very useful for the existing vineyards and, despite the fact that they are still not very commonly adopted, they are grabbing the attention of winegrowers. However, in a long-term perspective, a cultivar selection strategy could be the most sustainable adaptation method, as it reduces the management input requests. The collection and description of the existent grapevine biodiversity is the first step to achieving this goal, and some widespread collaborative works have demonstrated the general interest on this topic by viticulture researchers [34,35,36,37]. In this frame, it is worth noting the importance of the hot Mediterranean areas, a battlefront against the climate change impacts and a precious source of biodiversity for viticulture [38,39].

The aim of this study is to (i) describe the winter warming during the last decades in Salento in the perspective of its impact on viticulture, (ii) describe the anomalous budburst observed in the 2019–2020 winter, (iii) highlight the systemic effect of the budburst on the entire vine, and (iv) identify promising genotypes among ancient varieties grown in an old vineyard in Salento as a precious source of biodiversity to be further investigated in future studies.

## 2. Materials and Methods

### 2.1. Plant Material and Experimental Design

**Figure 1:**
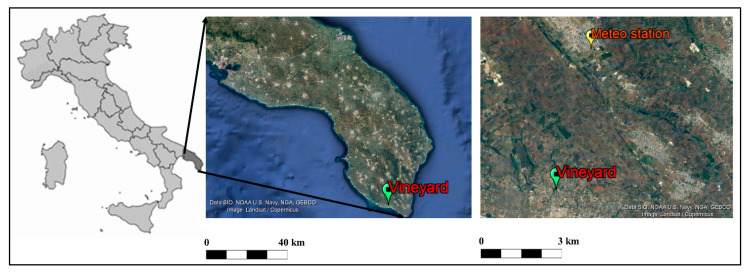
Map of Italy showing the Salento area (dark grey) in the Apulia Region and satellite images (from Google Earth) showing the position of the old vineyard (study area) and Meteo station.

The experiment was carried out in an old small vineyard (355 vines) located in Pescoluse, Salento (Italy) (Lat.: 39°50′45″ N, Long.: 18°15′13″ E, Alt.: 39 m a.s.l.) (Figure 1). In 1976, rooted cuttings of 157-11 Couderc rootstocks were planted in the field and then they were grafted *in loco* with buds collected through mass selection in an adjacent vineyard that was already old in that period. At the end of December 2019, in the middle of wintertime, the winegrower observed a bud break, and then, on 11 January 2020, a site inspection was organized to record the phenological stage of each bud developed on the principal shoots in 266 plants (3/4 of the total number of plants grown in this field). A total of 40,707 buds were observed. Samples of 1-year-old lignified shoots were also collected from a subgroup of plants (132 vines) to be analyzed concerning the starch content and the stem humidity.

Then, during the late wintertime, vines were normally pruned (training system: goblet).

On 18 May 2020, another site inspection was carried out to perform an ampelographic description of all the vines grown in the vineyard. Plants were classified based on their homogeneous phenotype and on the winegrower experience in eight cultivars (Baresana, Negroamaro, Malvasia nera di Brindisi, Malvasia bianca lunga, Seyve Villard 12.375, Bianco Palmento, Uva Gerusalemme, and Sprino). These genotypes were also identified by SNP analysis.

On 21 August 2020, a final site inspection was organized to complete the ampelographic description on grape bunches.

### 2.2. Meteorological Data and Analysis

Meteorological data were obtained from the station located in Presicce (Province of Lecce in the Salento area) (Figure 1) and managed by the Sezione Protezione Civile Regione Puglia—Centro Funzionale Decentrato di Modugno. It is located approximately 6 km from the experimental vineyard. Daily temperatures (minimum and maximum) were available since 1951, allowing a comprehensive study of the climatic variations that occurred during the last decades in the area. To achieve this goal, two 30-year periods were compared: 1961–1990 and 1991–2020.

In order to understand the relation between grapevine development and active temperature, growing degree days with 6 °C of base temperature [39,40,41,42] were calculated since November 1st. The chilling requirements for endodormancy were not considered. This choice was justified by the observation of early winter bud breaks.

The average monthly temperature (maximum and minimum), as well as the cumulative temperatures for active growth to overcome ecodormancy of the studied season, were compared with the climate normal 1961–1990 and 1991–2020. The two normal represent past and current climate, due to the shift in temperature regimes observed in Europe at the end of the 80s. The occurrence of frost events was also analyzed (monthly number of days with minimum temperature below 0 °C).

### 2.3. Plant Phenotyping

Plant phenology was recorded on 11 January 2020, considering each bud of the principal shoots by using the BBCH (“*Biologische Bundesanstalt, Bundessortenamt und CHemische Industrie*”) scale [43]. In 266 plants, a total of 40,707 buds were observed.

In the subgroup of 132 vines, starch content was measured in 5 biological replications for each considered plant (for a total of 660 analyses), following the method reported in [44]. Briefly, woody slides were obtained by using a penknife. Starch–iodine complexation was obtained by reacting a drop of Lugol solution for 3 min. Reflectance spectra were collected using a Jaz System spectrometer (Ocean Optics, B.V., Dunedin, FL, USA) set up as described in [45]. The starch index was calculated using the following formula:

$$INDEX_{\mathrm{Starch}-\mathrm{Iodine}\;\mathrm{Complex}}=1/\left(\frac{R_{Re\left(555\right)}}{R_{Re\left(900\right)}}\right)-1/\left(\frac{R_{t0\left(555\right)}}{R_{t0\left(900\right)}}\right)=\frac{R_{Re\left(900\right)}}{R_{Re\left(555\right)}}-\frac{R_{t0\left(900\right)}}{R_{t0\left(555\right)}}$$

*Rx(w) =* Reflectance (% calibrated on the reference blank)
*x =* spectrum type: t0 = before reaction; *Re* = after Lugol reaction
*w =* wavelength of interest (nm): 900 = normalization reference; 555 = starch–iodine complex absorption maximum.

Considering the same 132 vines, the shoot humidity was measured on 6 samples/plant (3 nodes and 3 internodes, for a total of 792 samples; clippings of about 2 g). Samples were weighed (Mwet), dried in an oven at 105 degrees for 24 h, and then weighed again (Mdry). The humidity was calculated by using the formula:

Shoot humidity=100×(Mwet−Mdry)Mwet

Ampelographic description was carried out using the “Manual for standardization of *Vitis* descriptors” [46] during spring- and summertime. A total of 38 ampelographic characters were described for each accession.

Statistical analyses were carried out using SPSS. For the 132 subgroup plants, it was calculated, for each vine, the maximum, average, and standard deviation BBCH recorded on 11 January 2020. Pearson’s correlations were calculated among these variables, the shoot humidity, and the starch content.

Regarding the total of 266 plants observed concerning phenology, the average BBCH of the buds/shoots of each plant was used to perform an analysis of variance (ANOVA), followed by Duncan’s post hoc test, to compare the cultivar response to winter budding.

### 2.4. SNP Genotyping

Genomic DNA of the eight cultivars studied was extracted from 0.1 mg of dried young leaves (1–2 cm diameter) using NucleoSpin^®^ Plant II (MACHEREY-NAGEL, Düren, Germany) according to the manufacturer’s protocol. DNA quality (260/230 and 260/280 ratios) was checked with a NanoDrop spectrophotometer (Thermo Fisher Scientific, Waltham, MA, USA), DNA degradation was verified by an electrophoresis on 1% agarose gel stained with ethidium bromide, and DNA concentration was detected by Quant-iT dsDNA HS (High Sensitivity) assay kit for Qubit 3.0 Fluorometer (Thermo Fisher Scientific, Waltham, MA, USA). SNP genotyping was performed on 200 ng of genomic DNA using the Vitis18kSNP genotyping array (Illumina Inc., San Diego, CA, USA) [37], covering 18,071 SNP loci. Genotyping was performed by the laboratory of Fondazione Edmund Much (San Michele all’Adige, Trento, Italy), according to the manufacturer’s instruction.

SNP profiles were filtered for call quality (p50GC) and GenTrain (GT) values. Samples with a p50GC value lower than 0.54 and loci with a GT score value lower than 0.6 were removed, as well as those with more than 20% missing data and monomorphic loci [47]. In order to ascertain the true-to-typeness of each analyzed cultivar, the filtered SNP profiles were compared with two already published datasets, reported in [38] and [39] and holding SNP profiles of about 1000 unique genotypes coming from Italy, Europe, and other wine growing areas, and a dataset [48] holding SNP profiles of about 400 unique genotypes coming from Italy.

## 3. Results

### 3.1. The Winter Warming

The comparison between average values of the 1961/1990 and 1991/2020 normals highlights the warming of the current climate phase (Table 1a–c), as confirmed by the pattern of monthly average minimum and maximum temperature, showing a significant increase in November, March, and April in the case of minimum temperature and in all the six months for maximum temperatures.

As a consequence, the accumulation of thermal resources for grapevine growth increases significatively in November, March, and April. Inversely, the period of November–January is stationary because of temperatures below the 6 °C threshold adopted for the calculation of active temperatures (see also supplementary material—Figures S1 and S2).

**Figure 2:**
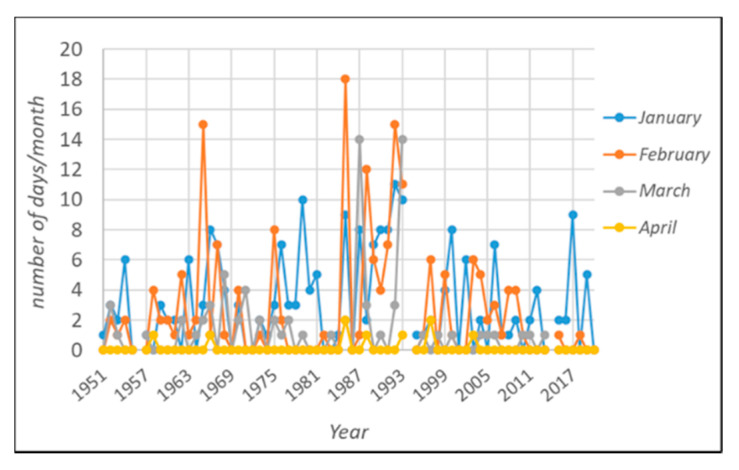
Frost risk evaluation: number of days/month reaching minimum temperatures lower than 0.

Regarding the occurrence of frost events, the analysis of the time series (Table 1d and Figure 2) shows no significant trend, with the only exception being November. Frosts are strongly related to specific circulation patterns and show a high interannual variability. Focusing on the 2019/2020 period, the winter season can be considered warm, even in relation to the current climate phase, with all the considered indicators showing values higher than the average for all the months except April (Table 1a–c). In more detail, the accumulation of thermal resources overcame the 1991/2020 average by 26.2, 43.3, 23.8, and 32.2% in the November–February period. March and April were characterized by negligible variation when compared with the normal (+5.5% and −3.4%, respectively).

Regarding the monthly occurrence of frost events, 2019/2020 did not face any case of frost.

### 3.2. The Winter Budding Concerns the Entire Plant

**Figure 3:**
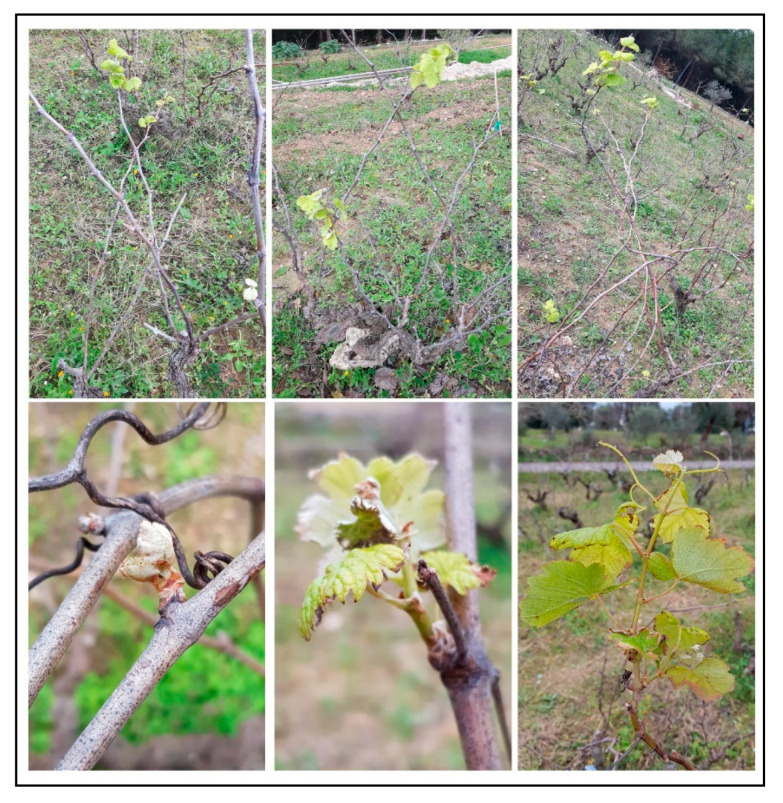
Photos of the bud break observed on 11 January 2020.

Photos of the budburst observed on 11 January 2020 are available in Figure 3.

BBCH values ranged from 0 (dormancy: winter buds pointed to rounded, light or dark brown, and bud scales more or less closed according to cultivar) to 17 (seven leaves unfolded and spread away from shoot). Significant Pearson’s direct correlations were observed among different phenology variables (maximum BBCH, average BBCH, and BBCH standard deviation), as well as with the shoot humidity of each plant. The starch content showed an indirect significant correlation with all the other variables (Table 2).

As expected, a strong acrotonic effect was observed. Therefore, the phenological inhomogeneity among buds increased in the sprouted plants, as demonstrated by the significant correlation among the BBCH standard deviation with both the BBCH average and maximum. The plant budburst resulted in a shoot hydration and in a consumption of the carbon reserves of the plant.

### 3.3. Old Vineyards: A Source of Biodiversity

The eight cultivars analyzed in this work were genotyped using the Vitis18kSNP genotyping array in order to ascertain their true-to-typeness. No samples showed a p50GC value lower than 0.54, while 1767 out of 18,071 loci (around 10%) had a GT score value lower than 0.6 and were removed. The number of monomorphic loci accounted for 4785 SNPs, while the number of loci showing a percentage of missing data higher than 20% was 100. After this filtering, each SNP profile was made up of 11,491 SNP loci (around 63% of loci included in the genotyping array) suitable for cultivar identification (Supplementary Table S1). By comparing the SNP profiles of the eight cultivars analyzed in this work with the ones (around 1400 SNP profiles) included in available datasets [38,39,48], the genetic profile of Baresana, Negroamaro, Malvasia nera di Brindisi, Malvasia bianca lunga, and Seyve Villard 12.375 varieties was confirmed. No SNP profile matched with the ones of Bianco Palmento, Uva Gerusalemme, and Sprino varieties.

**Figure 4:**
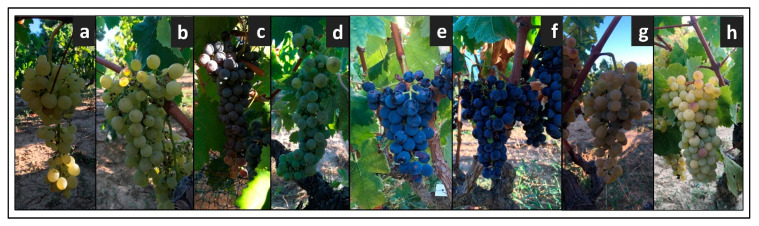
Bunches of the cultivars identified in the old vineyard: (**a**) Baresana, (**b**) Bianco Palmento, (**c**) Uva Gerusalemme, (**d**) Malvasia bianca lunga, (**e**) Malvasia near di Brindisi, (**f**) Negroamaro, (**g**) Seyve Villard 12.375, (**h**) Sprino.

Figure 4 reports the photos of the bunches of each cultivar, while the complete ampelographic description of these genotypes is available in supplementary materials (File S1).

### 3.4. Different Cultivars Have Different Phenological Behavior

**Figure 5:**
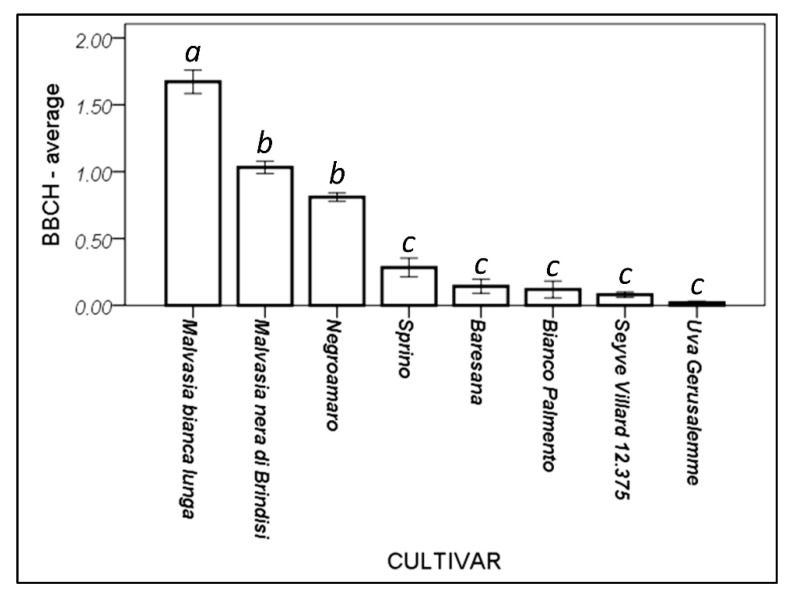
Cultivar response to winter budding. Higher values indicate a more advanced phenological phase. Bars indicate the standard error (±1). Different letters above the bars represent significantly different means (Duncan’s post hoc test, *p* = 0.05).

Different cultivars showed a different response to the warm winter conditions (Figure 5). Malvasia bianca lunga appeared to be the most susceptible to the anticipated sprouting, followed by Malvasia nera di Brindisi and Negroamaro. Beside the interspecific hybrid (Seyve Villard 12.375), Sprino, Baresana, Bianco Palmento, and Uva Gerusalemme—the minor autochthonous cultivars and the less commonly cultivated ones—appeared to better face the climatic changed conditions, avoiding the seasonal confusion caused by the global warming.

## 4. Discussion and Conclusions

The analysis of the thermal features of the area highlighted the general warming of winter that characterizes the current climatic phase. In this regard, the period 2019/2020 overcame the average values of the 1991–2020 normal, being characterized by a high availability of thermal resources for grapevine growth. Considering the relatively low chilling requirement of grapevine plants to overcome endodormancy (50–400 h at temperatures < 7 °C) [12], the warm temperature can cause the ecodormancy to be overcome early in winter, determining the anticipation of bud break.

The analysis of the time series shows no significant trends concerning the occurrence of frost events. This result is coherent with other analysis carried out in Europe [9,49,50]. Frosts are strongly related to specific circulation patterns and show a high interannual variability. This, together with the increase in active temperature availability and the consequent advance of phenology, exposes grapevine to a higher risk of damage [40,50,51,52,53,54,55]. In fact, as underlined by several authors [14,51,56], the early start of the vegetative growth increases the risk of damages induced by spring frosts, due to the high susceptibility of the plants. In fact, the peculiar conditions of the 2019/2020 vintage that cannot be considered a singular event (see the maximum values recorded during the last decades and the standard deviation of the data) resulted in an early and anomalous bud break.

The physiological dysfunctions caused by these adverse meteorological conditions (e.g., short exposure to chilling), together with the acrotony effect, resulted in a very high variability in the bud break along the shoots. The low exposure to chilling temperatures could also have decreased the number of buds involved in the anticipated sprouting [57]. Generally, these effects allowed the preservation of the correct phenological stage in the basal buds. This protective mechanism of the productive buds has also been used by researchers to postpone the plant phenology through late or double pruning [23,24,25,26,27].

It is worth noting that the observed sprouting is not limited to the singular buds susceptible to undesired growth; our data demonstrated the systemic response of the plants to the environmental conditions (e.g., soil temperature and water availability). In fact, the advanced phenological stages were related to stem hydration and consumption of the starch reserves characteristic of this phenological phase [58]. Of course, the carbohydrate reserve consumption post-budbreak will depend on the number of developed buds on the non-pruned winter cane, and on the post-budbreak pruning dates. The expected consequence of these events is at least a decrease in the production yield, but it could also affect the grape quality [25,28,29,30,31,32,33]. Further experiments could be focused on the effects of the pruning dates on the carbohydrate reserve consumption in these cultivars after budbreak.

Luckily, genotype affects the plant phenology, and different grapevine cultivars could have significantly different phenological timing [35,59,60]. The old vineyard studied in this work showed a good intraspecific variability, due to the massive selection adopted for the in-field grafting. Among the eight cultivars identified, and excluding the interspecific hybrid, the less known cultivars and, thus, those with the highest risk of extinction appeared to be the most interesting in the perspective of a climate change. The life cycle of grapevine lasts many years. The selection of cultivars able to adapt their phenology to the climatic changing conditions becomes fundamental in the perspective of a sustainable agriculture.

Old vineyards are a precious source of intraspecific biodiversity, but they are becoming rare due to the homogeneity objectives of the modern viticulture that favor monoclonal vineyards. For these reasons, the protection of these sources of biodiversity should be considered a priority.

## Figures and Tables

**Table 1:** Climatic analysis of the experimental area. Significant differences are written in bold.

**(a) Minimum Temperatures**										
	**2019/2020**	**1991–2020**				**1961–1990**				**Significance of the Difference (1961–1990 vs. 1991–2020)**
		**Aver.**	**Min.**	**Max.**	**St. Dev.**	**Aver.**	**Min.**	**Max.**	**St. Dev.**	
November	12.31	10.0	7.1	12.5	1.6	8.6	4.8	11.5	1.6	0.007 **
December	8.96	6.3	1.0	9.7	1.9	5.7	0.9	9.6	1.6	0.184
January	5.75	4.7	1.9	7.8	1.8	4.3	1.8	7.5	1.5	0.576
February	6.10	5.1	0.4	9.0	2.0	4.6	0.3	7.8	1.7	0.295
March	7.52	7.3	2.6	10.6	1.7	6.2	1.7	8.4	1.5	0.007 **
April	9.87	10.1	5.7	12.8	1.6	8.8	7.2	10.8	0.9	0.000 ***
**(b) Maximum Temperatures**										
	**2019/2020**	**1991–2020**				**1961–1990**				**Significance of the Difference (1961–1990 vs. 1991–2020)**
		**Aver.**	**Min.**	**Max.**	**St. Dev.**	**Aver.**	**Min.**	**Max.**	**St. Dev.**	
November	19.56	17.7	15.4	19.9	1.2	16.4	13.3	18.7	1.1	0.001 ***
December	15.03	13.8	9.0	16.5	1.6	12.9	10.2	14.6	1.0	0.021 *
January	13.57	12.6	10.1	14.8	1.2	11.7	7.7	14.2	1.3	0.035 *
February	15.44	13.3	10.1	16.2	1.7	12.3	8.1	15.0	1.4	0.016 *
March	16.19	15.6	12.3	19.0	1.3	14.5	9.8	17.9	1.9	0.012 *
April	18.56	19.0	14.9	22.4	1.6	17.8	15.6	20.5	1.4	0.002 ***
**(c) Cumulative Active Temperatures**										
	**2019/2020**	**1991–2020**				**1961–1990**				**Significance of the Difference (1961–1990 vs. 1991–2020)**
		**Aver.**	**Min.**	**Max.**	**St. Dev.**	**Aver.**	**Min.**	**Max.**	**St. Dev.**	
November	482.90	382.7	293.6	491.9	62.3	324.6	164.6	429.0	62.5	0.003 ***
December	325.05	226.8	31.6	325.1	71.7	195.4	64.7	338.8	57.2	0.079
January	202.75	163.8	68.8	272.0	62.1	142.7	51.2	249.3	53.1	0.362
February	226.95	171.7	35.5	319.6	70.1	144.6	35.2	245.0	51.2	0.090
March	298.30	282.6	120.7	435.4	66.5	234.5	110.1	337.9	59.7	0.006 **
April	394.65	408.7	214.8	540.5	69.9	343.1	270.9	442.4	49.2	0.000 ***
**(d) Occurrence of Frost Events (Number of Days with Temperatures Lower than 0)**										
	**2019/2020**	**1991–2020**				**1961–1990**				**Significance of the Difference (1961–1990 vs. 1991–2020)**
		**Aver.**	**Min.**	**Max.**	**St. Dev.**	**Aver.**	**Min.**	**Max.**	**St. Dev.**	
November	0	0.0	0	1	0.2	0.3	0	2	0.6	0.018 *
December	0	2.0	0	11	3.1	2.2	0	15	3.3	0.811
January	0	3.1	0	11	3.5	3.5	0	10	3.2	0.758
February	0	2.6	0	15	3.7	3.0	0	18	4.7	0.820
March	0	0.9	0	14	2.7	1.6	0	14	2.7	0.377
April	0	0.1	0	2	0.4	0.1	0	2	0.4	0.901

Aver. = Average, Min. = Minimum, Max. = Maximum, St. dev. = Standard deviation. (* *p* < 0.05; ** *p* < 0.01; *** *p* < 0.001).

**Table 2:** Pearson’s correlations among phenological parameters, shoot humidity, and starch content. All the obtained Pearson’s correlations have a 2-tails significance <0.000.

	BBCH Maximum	BBCH Average	BBCH Standard Deviation	Shoot Humidity	Starch Content
BBCH Maximum	1	0.796	0.876	0.393	−0.393
BBCH Average	0.796	1	0.888	0.562	−0.5
BBCH Standard Deviation	0.876	0.888	1	0.401	−0.457
Shoot humidity	0.393	0.562	0.401	1	−0.457
Starch content	−0.393	−0.5	−0.457	−0.404	−0.457

## Data Availability

The data presented in this study are available on request from the corresponding author.

## Supplementary Materials

The following are available online at https://www.mdpi.com/article/10.3390/foods10112769/s1, Figure S1: Maximum and minimum temperature trends during the period 1951–2020. Bars indicate the standard deviation of the records within the month. Red lines indicate the linear regression, Figure S2: Trends of the cumulative temperatures to overcome ecodormancy during the period 1951–2020. Red lines indicate the linear regression, Table S1: SNP profiles of eight cultivars, genotyped at 18k loci, File S1: Ampelographic description of the analyzed cultivars.
