# Supplementary material for: Climate Change Impacts on Plant Phenology: Grapevine (Vitis vinifera) Bud Break in Wintertime in Southern Italy
Source: Foods. 2021 Nov 11;10(11):2769. doi: 10.3390/foods10112769 (PMC8621361; doi:10.3390/foods10112769)
Supplement: Supplementary file 1 [file foods-10-02769-s001.zip › Figures S1 and S2.pdf]

SUPPLEMENTARY MATERIALS

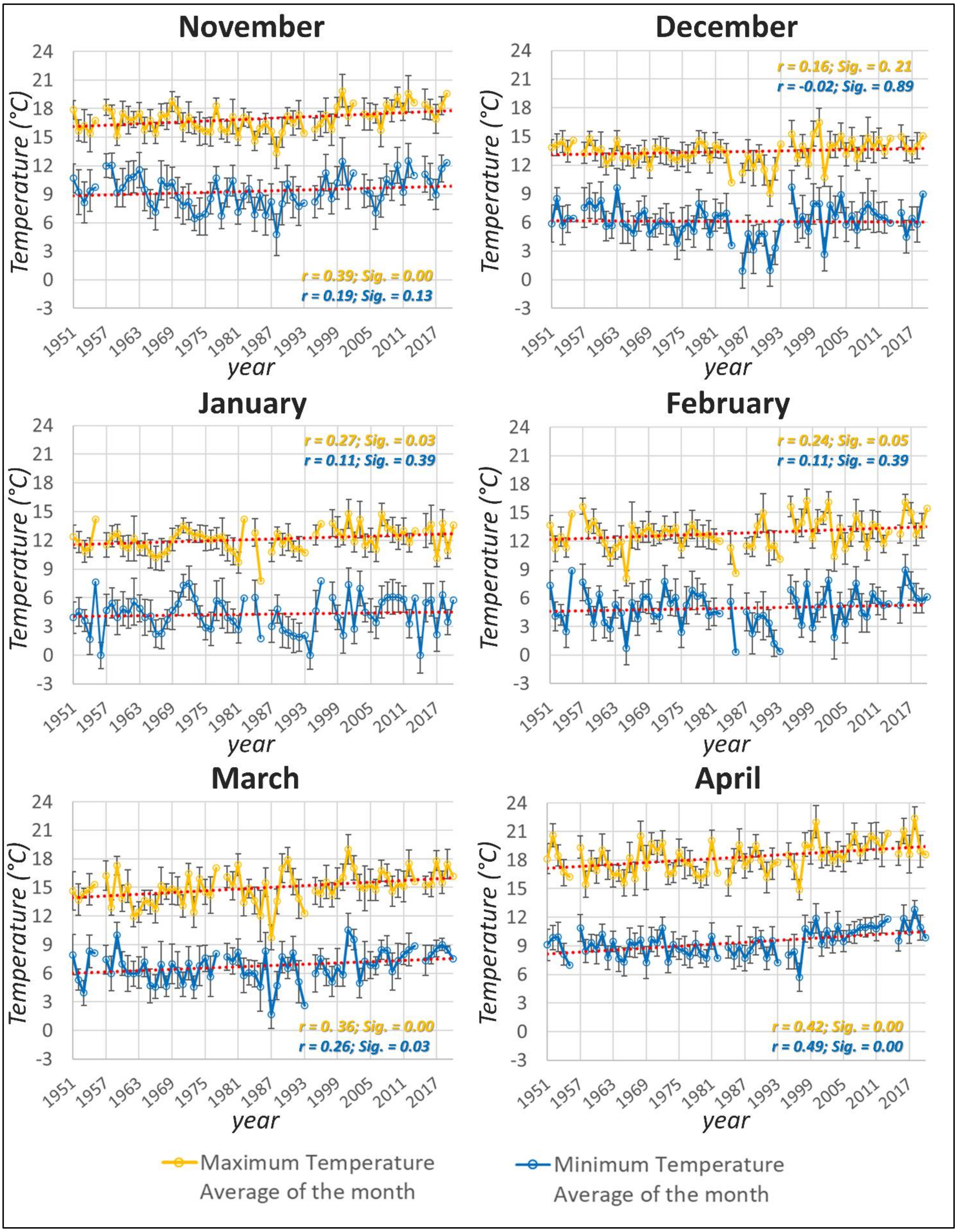

Figure S1: Maximum and minimum temperature trends during the period 1951-2020. Bars indicate the standard deviation of the records within the month. Red lines indicate the linear regression.

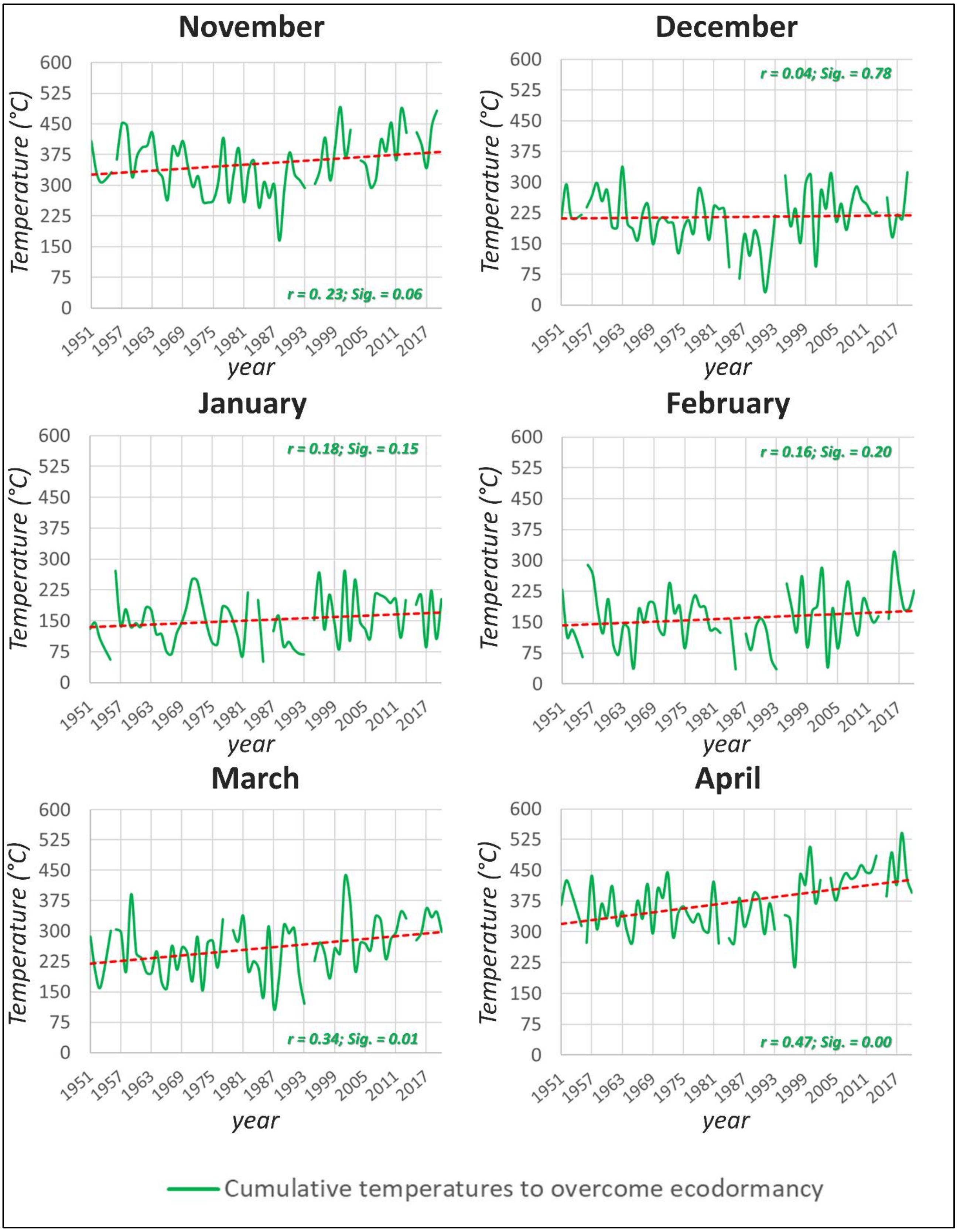

Figure S2: Trends of the cumulative temperatures to overcome ecodormancy during the period 1951-2020. Red lines indicate the linear regression.
