# Supplementary material for: Climate Change Impacts on Plant Phenology: Grapevine (Vitis vinifera) Bud Break in Wintertime in Southern Italy
Source: Foods. 2021 Nov 11;10(11):2769. doi: 10.3390/foods10112769 (PMC8621361; doi:10.3390/foods10112769)

BARESANA

The young shoot is fully open, with piping distribution of anthocyanin coloration on prostrate hairs of tip, with medium intensity of anthocyanin coloration on prostrate hairs on tip and medium to high density of prostrate hairs on tip, none or very low density of erect hairs on tip.
Concerning shoot characteristics, the color of dorsal side of internodes is green, color of the ventral sides of internodes and nodes is green and red, color of the dorsal side of nodes is green and red, none or very low density of erect and prostrate hairs on internodes, none or very low intensity of anthocyanin coloration on the bud scales.
The young leaf is copper-reddish; low density of prostrate hairs between main veins on lower side; none or very low density of erect hairs on main veins on lower side.
The mature leaf is large to very large, pentagonal, with five lobs. The area of anthocyanin coloration of main veins on upper side of blade reach the 1st bifurcation. It has strong goffering and medium-strong blistering. The teethes are both sides convex. The petiole sinus is strongly overlapped and V-shaped; the lateral sinuses are very shallow depth and open and V-shaped. Leaves have none or very low density of prostrate and erect hairs between main veins on lower side.
The flower is hermaphrodite with fully developed stamens and fully developed gynoecium.
Bunches are very long, between loose and very loose in berry density, with a cylindrical shape.
Berries are medium-long, with a broad ellipsoid shape, yellow green in color, without anthocyanins accumulation in the flesh, and with complete formation of seeds.

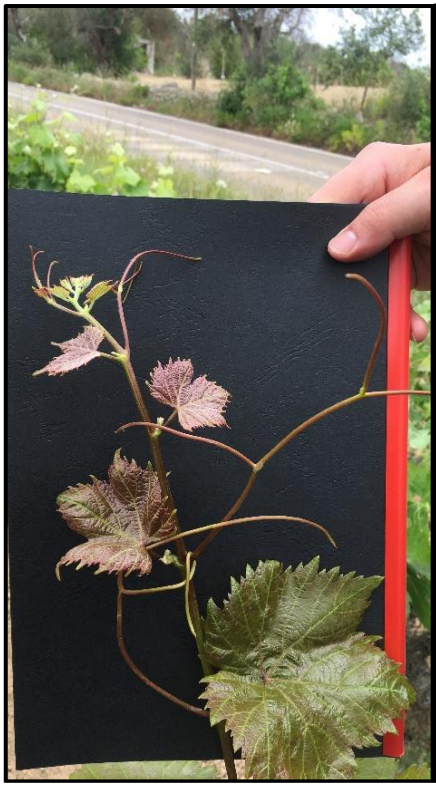
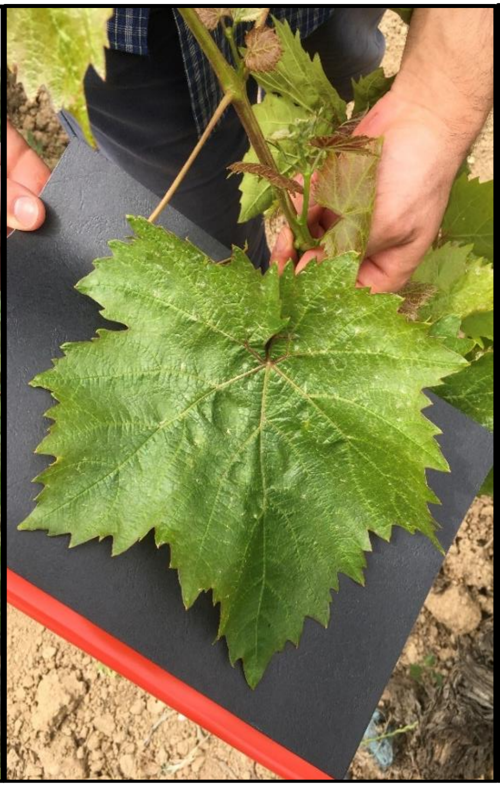
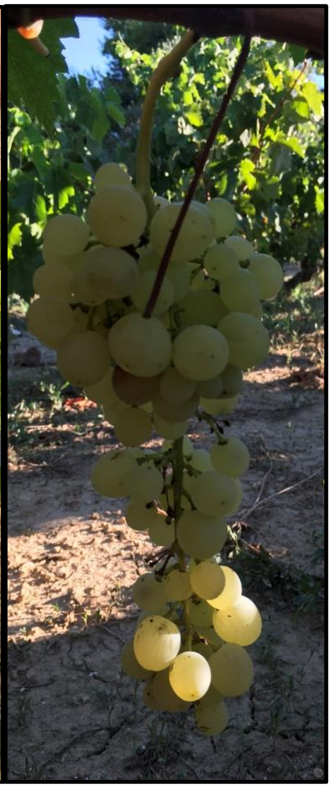

BIANCO PALMENTO

The young shoot is fully open, with absent distribution of anthocyanin coloration on prostrate hairs of tip, very high density of prostrate hairs on tip and low density of erect hairs on tip.
Concerning shoot characteristics, the color of dorsal and ventral sides of internodes and nodes is green and red, low density of erect hairs on internodes, medium density of prostrate hairs on internodes, none or very low intensity of anthocyanin coloration on the bud scales.
The young leaf is green, with high density of prostrate hairs between main veins on lower side and low density of erect hairs on main veins on lower side.
The mature leaf is large, circular, with five-seven lobs. The area of anthocyanin coloration of main veins on upper side of blade is limited to the petiolar point. It has medium goffering and strong blistering. The teethes are one side concave, one side convex. The petiole sinus is overlapped to strongly overlapped and U-shaped; the lateral sinuses are medium to deep depth, strongly overlapped and U-shaped. Leaves have high density of prostrate hairs between main veins on lower side and low density of erect hairs on the main veins on lower side.
The flower is hermaphrodite with fully developed stamens and fully developed gynoecium.
Bunches are long, between loose and very loose in berry density, with a conical shape.
Berries have medium length, with a broad ellipsoid shape, yellow green in color, without anthocyanins accumulation in the flesh, and with complete formation of seeds.

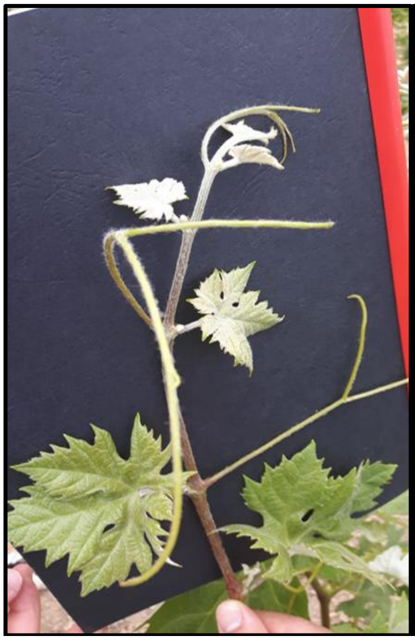
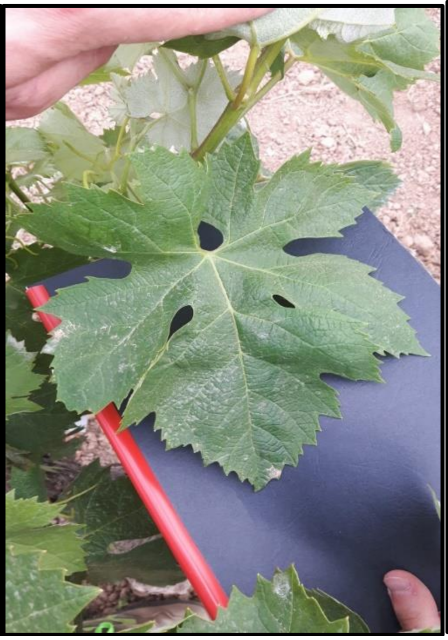
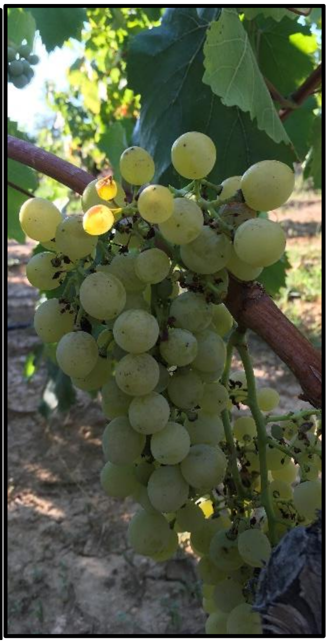

UVA GERUSALEMME

The young shoot is fully open, with absent anthocyanin coloration on prostrate hairs of tip, medium density of prostrate hairs on tip and low or very low density of erect hairs on tip.

Concerning shoot characteristics, the color of the dorsal sides of internodes and nodes is green and red, while the color of the ventral side of internodes and nodes is red, none or very low density of erect hairs on internodes, low density of prostrate hairs on internodes, strong intensity of anthocyanin coloration on the bud scales were recorded.

The young leaf is bronze, with none or very low density of prostrate hairs between main veins on lower side and medium to high density of erect hairs on main veins on lower side.

The mature leaf is medium sized, wedge-shaped, with three lobs. The area of anthocyanin coloration of main veins on upper side of blade reach the 2nd bifurcation. It has absent or very weak goffering and medium blistering. The teethes are one side concave, one side convex. The petiole sinus is open and brace-shaped; the lateral sinuses are shallow depth and open and U-shaped lateral sinuses. Leaves have none or very low density of prostrate and erect hairs on the main veins on lower side.

The flower is hermaphrodite with fully developed stamens and fully developed gynoecium.

Bunches are very long, loose in berry density, with a cylindrical shape.

Berries are medium-long, with a globose shape, grey red in color, without anthocyanins accumulation in the flesh, and with complete formation of seeds.

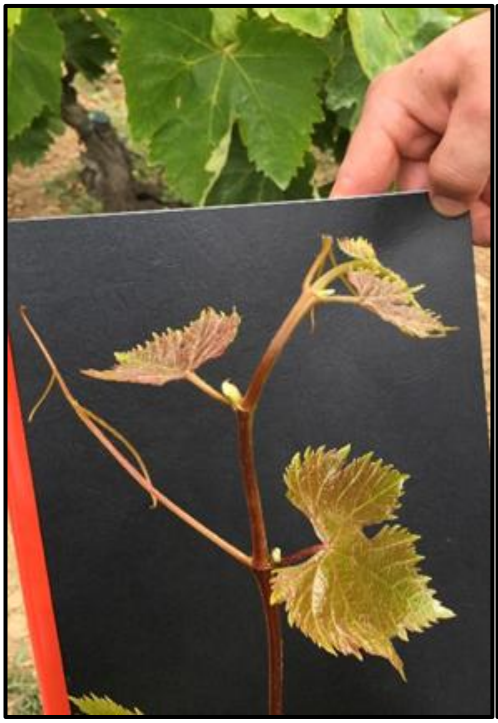
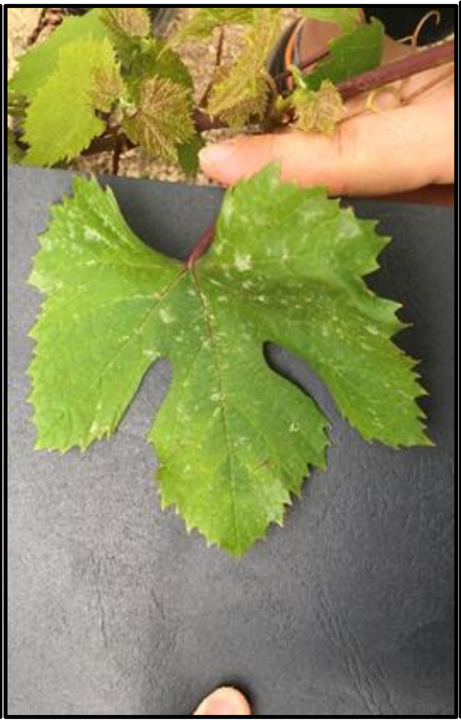
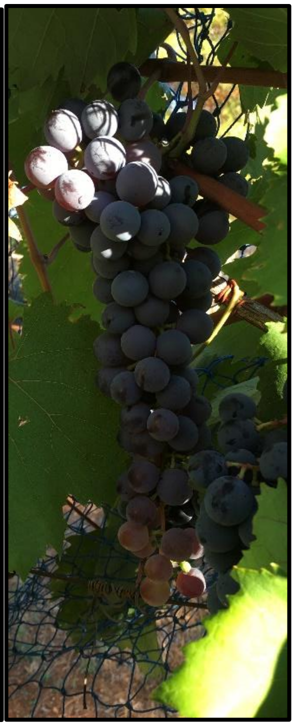

MALVASIA BIANCA LUNGA

The young shoot is fully open, without anthocyanin coloration on prostrate hairs, very high density of prostrate hairs on tip and medium density of erect hairs on tip.
Concerning shoot characteristics, the color of dorsal and ventral sides of internodes and nodes are green and red, none or very low density of erect and prostrate hairs on internodes, medium density of prostrate hairs on internodes, medium intensity of anthocyanin coloration on the bud scales.
The young leaf is green-light bronze; with very high density of prostrate hairs between main veins on lower side; none or very low density of erect hairs on main veins on lower side.
The mature leaf is medium, pentagonal, with seven lobs. The area of anthocyanin coloration of main veins on upper side of blade is limited to the petiolar point. It has weak goffering and medium blistering. The teethes are one side concave, one side convex. The petiole sinus is overlapped and U-shaped; the lateral sinuses have deep depth and they are strongly overlapped and U-shaped. Leaves have high density of prostrate hairs between the main veins and none or very low density and erect hairs on the main veins on lower side.
The flower is hermaphrodite with fully developed stamens and fully developed gynoecium.
Bunches are very long, between loose and medium berry density, with a cylindrical shape.
Berries are short, with a obloid shape, yellow green in color, without anthocyanins accumulation in the flesh, and with complete formation of seeds.

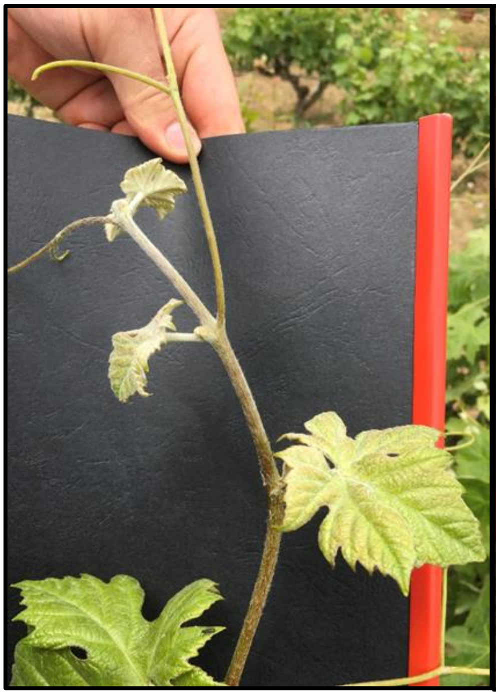
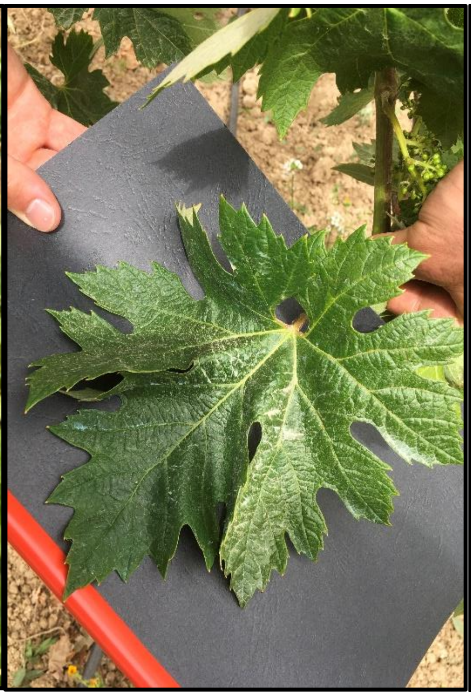
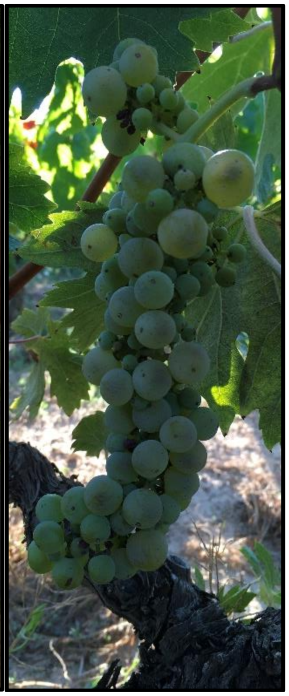

MALVASIA NERA DI BRINDISI

The young shoot is fully open, without anthocyanin coloration on prostrate hairs of tip, high density of prostrate hairs on tip, low density of erect hairs on tip.
Concerning shoot characteristics, the color of dorsal and ventral sides of internodes is green and red, none or very low density of erect hairs on internodes; low density of prostrate hairs on internodes; medium intensity of anthocyanin coloration on the bud scales.
The young leaf is green; with high density of prostrate hairs between main veins on lower side and none or very low density of erect hairs on main veins on lower side.
The mature leaf is medium to large, cordate, with five lobs. The area of anthocyanin coloration of main veins on upper side of blade is absent. It has absent or very weak goffering and weak blistering. The teethes are one side concave, one side convex. The petiole sinus is open and brace-shaped; the lateral sinuses are slightly overlapped, medium depth and U-shaped. Leaves have high density of prostrate hairs between main veins on lower side and low density of erect hairs on main veins on lower side.
The flower is hermaphrodite with fully developed stamens and fully developed gynoecium.
Bunches are medium-short, with a conical shape; they have a loose berry density.
Berries are short, with a globose-obloid shape, blue black in color, with a weak anthocyanin coloration in the flesh, and with complete formation of seeds.

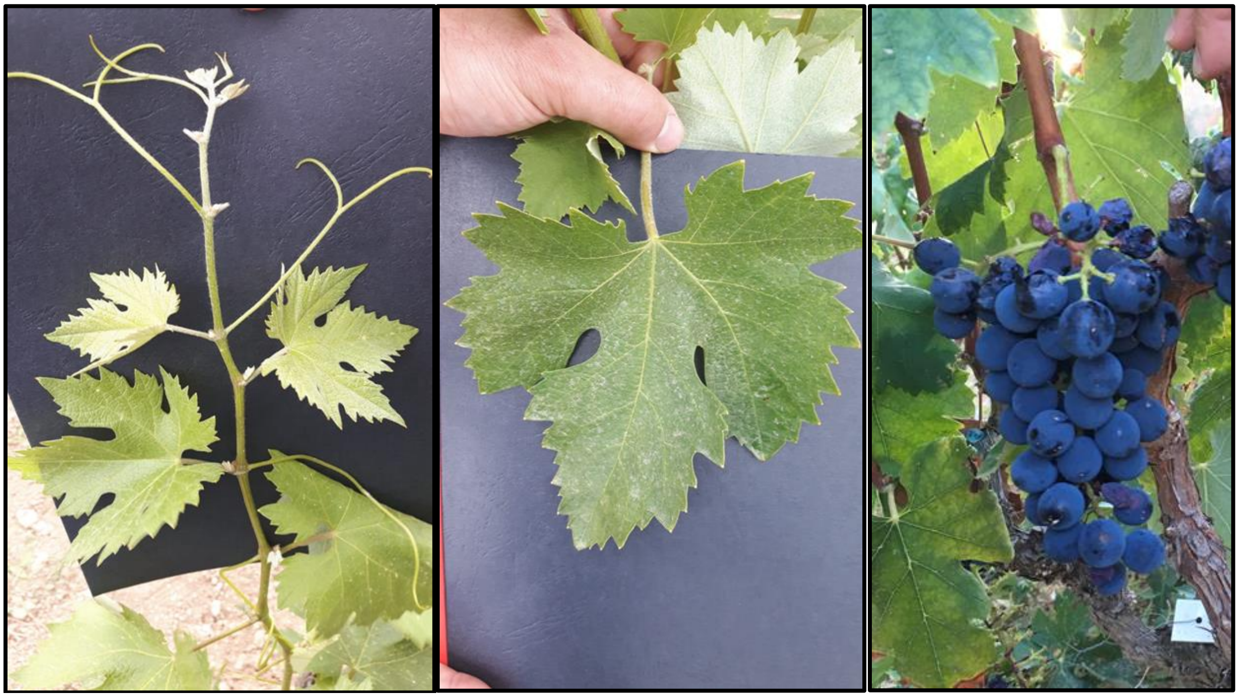

NEGROAMARO

The young shoot is fully open, with piping distribution of anthocyanin coloration on prostrate hairs of tip, low intensity of anthocyanin coloration on prostrate hairs on tip and very high density of prostrate hairs on tip, none or very low density of erect hairs on tip.

Concerning shoot characteristics, the color of dorsal and ventral sides of internodes and nodes is green, none or very low density of erect hairs on internodes; high density of prostrate hairs on internodes; none or very low intensity of anthocyanin coloration on the bud scales.

The young leaf is green; with high density of prostrate hairs between main veins on lower side and none or very low density of erect hairs on main veins on lower side.

The mature leaf is large, cordate, with three lobs. The area of anthocyanin coloration of main veins on upper side of blade reach only the petiolar point. It has weak goffering and medium blistering. The teethes are both sides convex. The petiole sinus is open and V-shaped; the lateral sinuses are deep depth and open and U-shaped. Leaves have high to very high density of prostrate hairs between main veins on lower side and low density of erect hairs on main veins on lower side.

The flower is hermaphrodite with fully developed stamens and fully developed gynoecium.

Bunches are short, with a conical shape; they have a medium berry density.

Berries are short or very short, with a globose shape, blue black in color, with a weak anthocyanin coloration in the flesh, and with complete formation of seeds.

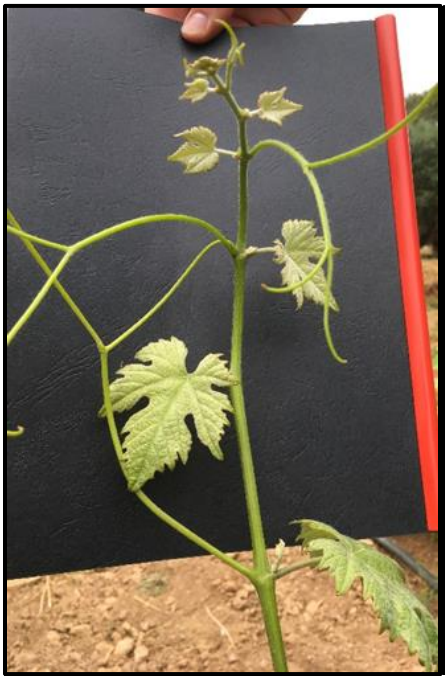
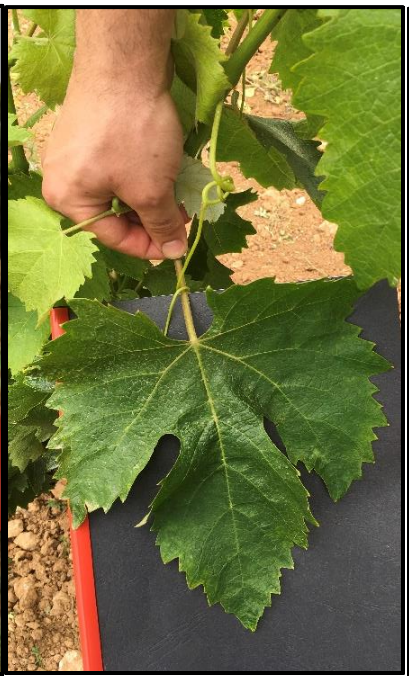
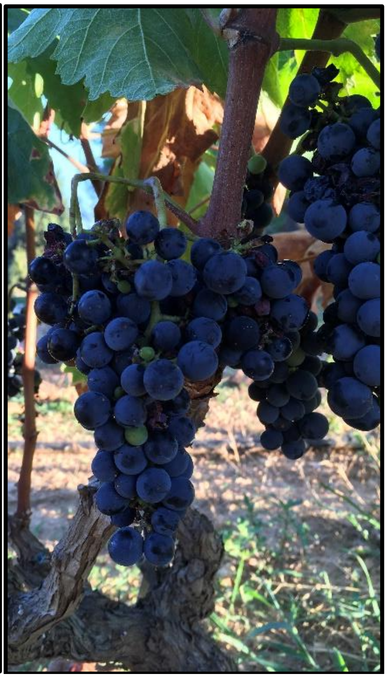

SEYVE VILLARD 12.375

The young shoot is half open, the blade of young leaves is pigmented, however, none ore very low density of prostrate and erect hairs on tip was observed, resulting in an absent distribution of anthocyanin coloration on prostrate hairs of tip.
Concerning shoot characteristics, the color of dorsal and ventral sides of internodes are green and red, the ventral side of the nodes is red, none or very low density of both erect and prostrate hairs on internodes, medium density of prostrate hairs on internodes, weak intensity of anthocyanin coloration on the bud scales.
The young leaf is bronze; with none or very low density of prostrate between main veins and of erect hairs on main veins on lower side.
The mature leaf is small, pentagonal, with five lobs. The area of anthocyanin coloration of main veins on upper side of blade reach the 1st bifurcation. It has weak goffering and weak blistering. The teethes are one side concave, one side convex. The petiole sinus is closed and U-shaped; the lateral sinuses are absent. Leaves have none or very low density of both prostrate hairs between the main veins and erect hairs on the main veins on lower side.
The flower is hermaphrodite with fully developed stamens and fully developed gynoecium.
Bunches are long, between loose and very loose berry density, with a conical shape.
Berries are short, with a globose shape, yellow green in color, without anthocyanins accumulation in the flesh, and with complete formation of seeds.

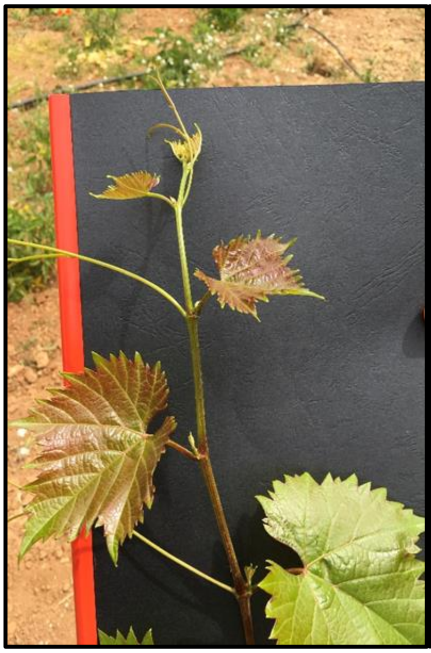
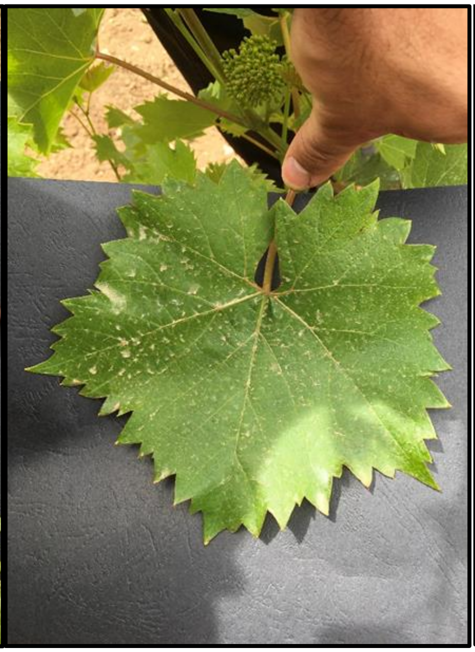
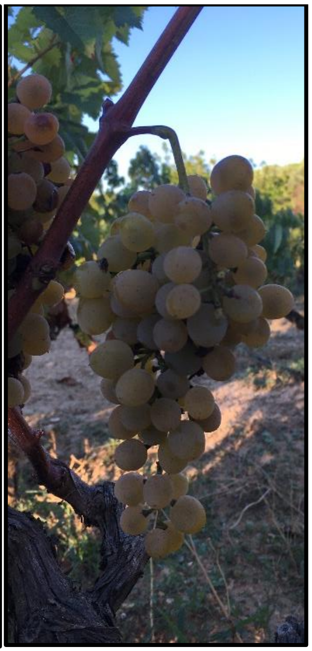

SPRINO

The young shoot is fully open, with absent anthocyanin coloration on prostrate hairs of tip, medium to high density of prostrate hairs on tip and none or very low density of erect hairs on tip.
Concerning shoot characteristics, the color of dorsal and ventral sides of internodes are green and red, while the color of dorsal and ventral side of nodes is red, none or very low density of erect hairs on internodes, low to medium density of prostrate hairs on internodes; medium to strong intensity of anthocyanin coloration on the bud scales.
The young leaf is bronze; with high density of prostrate hairs between main veins on lower side; none or very low density of erect hairs on main veins on lower side.
The mature leaf is medium sized, pentagonal, with five lobs. The area of anthocyanin coloration of main veins on upper side of blade is limited to the petiolar point. It has weak or absent goffering and medium blistering. The teethes are one side concave, one side convex. The petiole sinus is open and U-shaped; the lateral sinuses are slightly overlapped and U-shaped and have a medium low depth, with high variability among leaves. Leaves have high density of prostrate hairs between main veins on lower side and low density of erect hairs on main veins on lower side of blade.
The flower is hermaphrodite with fully developed stamens and fully developed gynoecium.
Bunches are very long, medium in berry density, with a cylindrical shape.
Berries are medium-short, with a broad ellipsoid shape, yellow green in color, without anthocyanins accumulation in the flesh, and with complete formation of seeds.

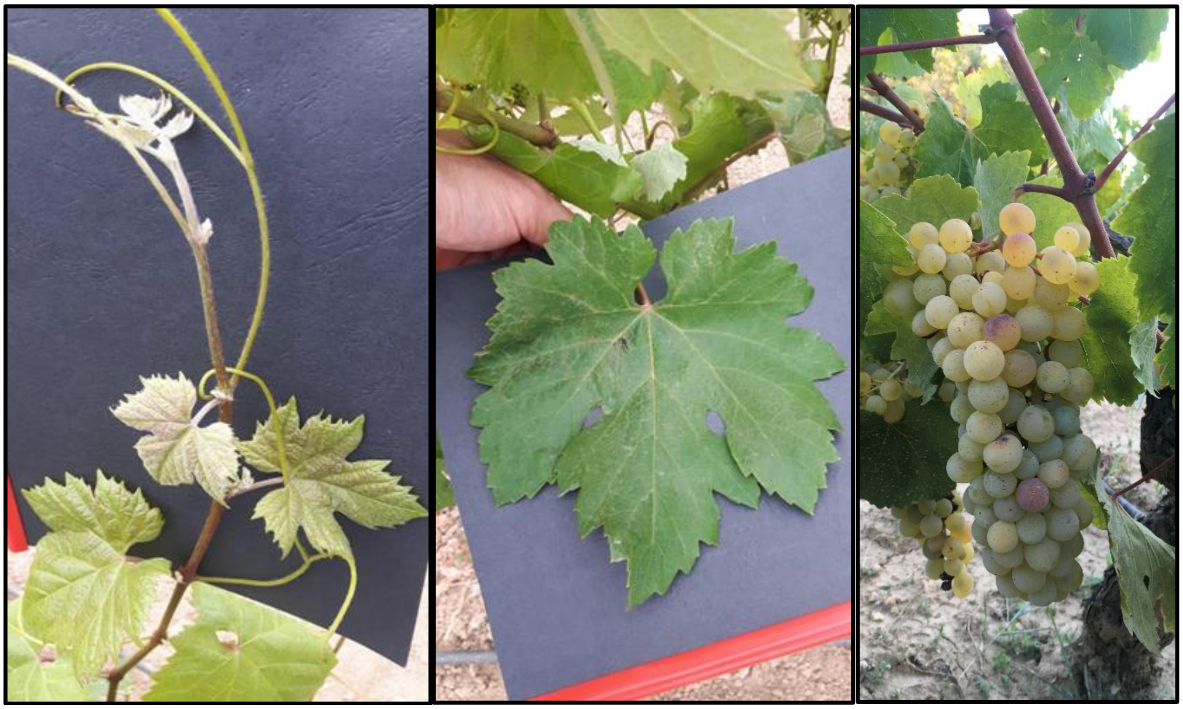

Supplement: Supplementary file 1 [file foods-10-02769-s001.zip › File S1.pdf]
